# The Impact of Exogenous Vitamin D on Pituitary Effects of Metformin in Postmenopausal Women with Subclinical Hypothyroidism and Normal Vitamin D Status: A Pilot Study

**DOI:** 10.3390/nu18050838

**Published:** 2026-03-05

**Authors:** Robert Krysiak, Karolina Kowalcze, Johannes Ott, Simona Zaami, Giuseppe Gullo, Bogusław Okopień

**Affiliations:** 1 Department of Internal Medicine and Clinical Pharmacology, Medical University of Silesia, Medyków 18, 40-752 Katowice, Poland; bokopien@sum.edu.pl; 2 Department of Pathophysiology, Faculty of Medicine, Academy of Silesia, Rolna 43, 40-555 Katowice, Poland; kkowalcze@sum.edu.pl; 3 Department of Pediatrics in Bytom, Faculty of Health Sciences in Katowice, Medical University of Silesia, Stefana Batorego 15, 41-902 Bytom, Poland; 4 Clinical Division of Gynecologic Endocrinology and Reproductive Medicine, Department of Obstetrics and Gynecology, Medical University of Vienna, 1090 Vienna, Austria; johannes.ott@meduniwien.ac.at; 5 Department of Anatomical, Histological, Forensic and Orthopedic Sciences, Sapienza University of Rome, 00161 Rome, Italy; simona.zaami@uniroma1.it; 6 Department of Obstetrics and Gynecology, Villa Sofia Cervello Hospital, University of Palermo, 90146 Palermo, Italy; gullogiuseppe@libero.it

**Keywords:** drug interactions, glucose homeostasis, gonadotropins, hypothalamic-pituitary-thyroid axis, menopause, metformin, other pituitary hormones, prolactin, vitamin D, women’s health

## Abstract

*Background/Objectives*: Low vitamin D status was found to attenuate the impact of metformin on circulating levels of anterior pituitary hormones, but this inhibitory effect was absent in vitamin D-repleted subjects. No previous study investigated the interaction between metformin and exogenous vitamin D at the pituitary levels in individuals with normal vitamin D status. *Methods*: Our pilot, single-center, prospective, matched-cohort study enrolled 59 postmenopausal women with subclinical hypothyroidism and 25-hydroxyvitamin D levels in the range between 75 and 150 nmol/L. For the following six months, all the participants were treated with either metformin/vitamin D combination therapy (group 1, *n* = 27) or metformin alone (group 2, *n* = 32). The outcomes of interest included 25-hydroxyvitamin D, fasting glucose, HOMA-IR, HbA_1c_, TSH, FSH, LH, prolactin, ACTH, free thyroid hormones, estradiol and IGF-1. A parallel study investigated the impact of vitamin D monotherapy on the outcome measures in insulin-resistant women meeting the remaining inclusion criteria. *Results*: No differences in baseline biomarker values were observed between groups 1 and 2. Ninety-three percent of the patients completed the study. The increase in 25-hydroxyvitamin D levels was observed exclusively in group 1. Although glucose homeostasis markers and post-treatment levels of TSH and FSH were lower at the end of the study than at baseline in both groups, the effect of treatment was more pronounced in group 1 than in group 2. Metformin/vitamin D combination therapy, but not metformin alone, reduced LH and prolactin levels. In both groups, the TSH- and gonadotropin-lowering effects of metformin correlated with baseline levels of these pituitary hormones. Levels of ACTH, free thyroxine, free triiodothyronine, estradiol and IGF-1 remained stable throughout the study. The effects of vitamin D monotherapy were confined to an increase in plasma 25-hydroxyvitamin D concentrations and a modest enhancement in insulin sensitivity. *Conclusions*: Exogenous vitamin D potentiates the pituitary effects of metformin in postmenopausal women with subclinical hypothyroidism.

## 1. Introduction

Owing to its proven efficacy and favorable safety profile, metformin is considered the first-line therapy for type 2 diabetes and is widely prescribed for other insulin-resistant conditions [1]. Beyond improving glucose homeostasis, the drug has been reported to have pleiotropic effects, including favorable effects on endothelium, anti-oxidative and anti-inflammatory properties, suppression of neoplastic cell growth, alteration of gut microbiota composition and function, and hormonal effects [2,3]. Findings from both meta-analyses [4,5,6] and smaller clinical studies [7,8] indicate that metformin lowers elevated levels of anterior pituitary hormones. The inhibitory effect on thyroid-stimulating hormone (TSH) in individuals with hypothyroidism [4,5] and prolactin in subjects with hyperprolactinemia [6] was independent of the underlying cause. In turn, decreases in gonadotropin levels have been reported in postmenopausal women and in men with primary hypogonadism [7,8]. This latter effect has been confirmed in a rat study assessing pituitary secretion of follicle-stimulating hormone (FSH) and luteinizing hormone (LH) after exposure to secretagogues [9]. Importantly, metformin, even at high doses, does not seem to affect circulating levels of anterior pituitary hormones if their pre-treatment concentrations are within the reference range [4,5,7,8]. Thus, the drug does not pose a risk of hypopituitarism and may be safely used in patients with normal pituitary function.

Recent studies conducted by our research group revealed that the impact of metformin on TSH, prolactin, and FSH was also absent in individuals with oversecretion of these hormones who were vitamin D-deficient or -insufficient. However, vitamin D deficiency does not appear to affect the pituitary actions of metformin when vitamin D homeostasis is restored [10,11,12]. Unfortunately, the studies’ design does not allow assessing whether vitamin D preparations modulate the impact of metformin on anterior pituitary hormones in individuals with normal vitamin D homeostasis. Answering this question would be interesting because exogenous vitamin D supplementation has been shown to reduce elevated glucose levels, decrease insulin resistance, improve plasma lipids (low- and high-density lipoprotein cholesterol and triglycerides), lower blood pressure (both systolic and diastolic), and protect against osteoporosis not only in patients with low vitamin D status but also in subjects whose vitamin D status was not determined [13,14,15,16]. Postmenopausal women appear to derive benefits from the co-administration of metformin and exogenous vitamin D. Menopausal transition is often complicated by excessive body weight, central fat distribution, hyperglycemia, insulin resistance, increased risk of diabetes and hypertension, and accelerated bone loss [17,18,19]. Owing to the relatively high prevalence of type 2 diabetes, prediabetes and metabolic syndrome in middle-aged and elderly women, [20] postmenopausal women frequently receive chronic metformin treatment. Lastly, administration of vitamin D is commonly recommended to prevent or delay the onset of postmenopausal osteoporosis and osteopenia [21].

Thus, the aim of this study was to assess the impact of exogenous vitamin D on pituitary effects of metformin in postmenopausal women with subclinical hypothyroidism and normal vitamin D status. Besides the reasons mentioned above, this population was selected because of the simultaneous elevation of several anterior pituitary hormones, including TSH, FSH, LH, and occasionally prolactin.

## 2. Materials and Methods

This was a pilot, single-center, prospective, matched-cohort study. The protocol adhered to the tenets of the 1975 Declaration of Helsinki for research involving human subjects and was approved by the Institutional Review Board of the Medical University of Silesia. Informed, written consent was provided by all participants prior to enrollment. Patient recruitment took place between May 2022 and January 2025. The study was conducted at the Department of Internal Medicine and Clinical Pharmacology, Medical University of Silesia, Katowice, Poland. Given the nature of the study (a cohort study), registration in a clinical trial registry was not necessary.

### 2.1. Participants

**Figure 1:**
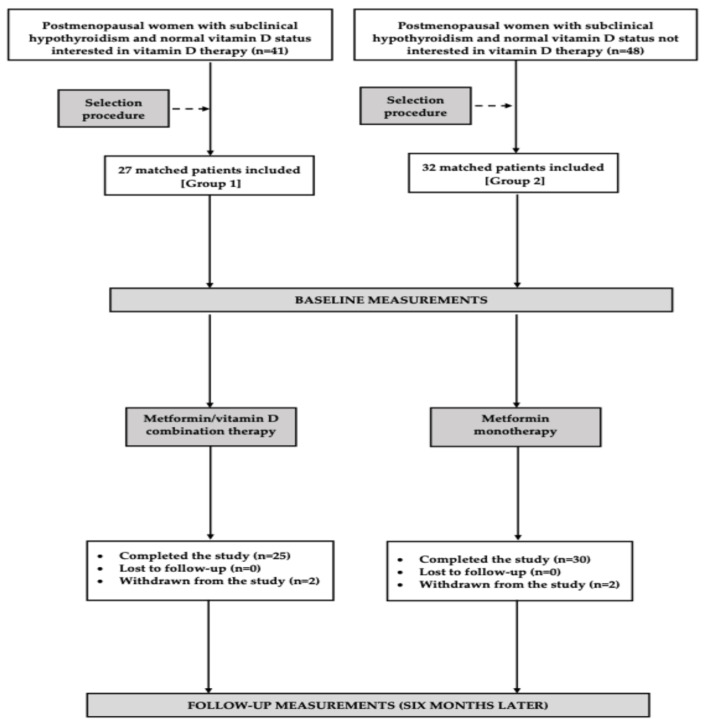
The flow of patients through the study.

This study enrolled two cohorts of postmenopausal women with untreated non-autoimmune subclinical hypothyroidism who qualified for metformin therapy owing to insufficient metabolic control of type 2 diabetes or prediabetes despite adherence to lifestyle interventions for a minimum of 12 weeks. Eligibility was restricted to individuals with adequate vitamin D status, defined as plasma 25-hydroxyvitamin D (25OHD) concentrations between 75 and 150 nmol/L. Postmenopausal status was defined as amenorrhea lasting at least 12 months, in conjunction with FSH levels > 30 U/L and plasma estradiol concentrations < 30 pg/mL (110 pmol/L). Non-autoimmune subclinical primary hypothyroidism was diagnosed based on the following criteria: circulating TSH concentrations of 4.5–10.0 mU/L; free thyroxine levels of 10.0–21.3 pmol/L; free triiodothyronine levels of 2.3–6.5 pmol/L; absence of circulating antibodies against thyroid peroxidase, thyroglobulin, and the thyrotropin receptor; and lack of ultrasonographic features indicative of autoimmune thyroid disease. Diabetes and prediabetes were diagnosed based on commonly accepted criteria [22]. The participants were allocated to one of two treatment groups: group 1 received metformin in combination with vitamin D (*n* = 27), whereas group 2 received metformin monotherapy (*n* = 32). Treatment allocation was based on patient preference. The sample size estimation indicated that a minimum of 23 participants per group was required to detect a 20% intergroup difference in TSH levels (the primary outcome measure) with a statistical power of 80% and a two-sided α level of 0.05. To account for potential withdrawals and losses to follow-up, the enrolled study population exceeded the calculated minimum sample size. To partially mitigate initial inclusion bias, only a subset—not all—of the individuals who met the inclusion and exclusion criteria and consented to participate and receive metformin, with or without vitamin D, were enrolled (Figure 1). Of the 89 individuals eligible for metformin, 41 chose to take exogenous vitamin D, while 48 did not. The potential participants underwent a selection procedure using the PEPI-for-Windows computer program, version 3.26 (Brixton Health, Llanidloes, UK), to ensure that the two cohorts were closely matched for age, fasting glucose, 25OHD levels, and TSH levels, thereby minimizing baseline differences. To minimize the influence of seasonal variability on plasma 25OHD concentrations [23], comparable numbers of participants were enrolled during the winter months (December–January; 12 in group 1 and 17 in group 2) and during late spring/early summer (May–June; 15 in each group).

The exclusion criteria comprised HbA_1c_ (glycated hemoglobin) levels exceeding 8.0%; the presence of other endocrine disorders; cardiovascular disease; a glomerular filtration rate below 60 mL/min/1.73 m^2^; any elevation in total bilirubin and/or transaminase levels; anemia; malabsorption syndromes; chronic inflammatory conditions; other significant comorbidities; use of hormone replacement therapy or other concurrent hormonal treatments; concomitant use of additional antidiabetic agents; and treatment with medications known to interact with metformin or vitamin D.

### 2.2. Study Design

In the first week of treatment, metformin was administered at a dose of 500 mg twice daily. The dosage was subsequently titrated in a stepwise manner to a target daily dose of 2.55–3.0 g, which was achieved by week 4 and maintained for the remainder of the study period. The target dose was administered in three divided doses. To mitigate the risk of gastrointestinal adverse effects, metformin was taken with or immediately following meals. Tablets were ingested whole and were neither crushed nor chewed. Additionally, the participants assigned to group 1 received oral vitamin D in capsule form, administered once daily in the morning at a dose of 2000 IU (50 μg). Dietary intake of vitamin D was evaluated using individual dietary questionnaires assessing the frequency and quantity of consumption of the 20 most commonly consumed dishes in Polish cuisine over the preceding two weeks. Mean daily intake of these foods was calculated, multiplied by their respective vitamin D content, and aggregated to estimate total dietary vitamin D intake. Nutrient composition data were derived from food composition tables developed by the Polish National Food and Nutrition Institute in Warsaw [24]. The short-term use of prescription or over-the-counter medications (≤7 days) was permitted only if such treatment had been discontinued at least four weeks prior to study completion. Participants were instructed to maintain their habitual lifestyle behaviors throughout the study. Adherence to metformin therapy was assessed via pill counts and participant self-reporting, with satisfactory compliance defined as ingestion of at least 90% of the prescribed doses. The withdrawal criteria included the occurrence of serious adverse events (as defined by the Food and Drug Administration) [25], changes in pharmacological treatment other than those specified above, failure to adhere to the study protocol, and withdrawal of informed consent.

### 2.3. Parallel Study

Because patients with type 2 diabetes and many individuals with prediabetes require disease-specific therapy, the use of vitamin D monotherapy without hypoglycemic treatment was considered unethical. To evaluate whether vitamin D monotherapy influenced the study findings, a separate parallel analysis was performed in a cohort of 25 postmenopausal women with insulin resistance, non-autoimmune subclinical hypothyroidism and normal vitamin D status who were not included in the primary investigation. All the participants followed the prescribed lifestyle intervention, declined antidiabetic medication, and were monitored by the research team over the same time frame as the other study groups.

### 2.4. Laboratory Assays

Venous blood was collected from each patient by antecubital venipuncture before and at the end of the study period. To standardize this procedure, all the subjects fasted for at least 12 h, and blood was taken at 8 a.m. Prior to sample collection, each individual had remained at rest in a seated position for at least half an hour. To ensure objective laboratory assessments by minimizing performance bias, the measurements were performed by a technician blinded to the aims of the study and patient group assignment. All samples were run in duplicate to confirm the accuracy and reproducibility of the measurements. Prolactin concentration was assessed in three blood samples taken at 20 min intervals. Plasma concentrations of glucose and HbA_1c_ in the whole blood were measured using the COBAS Integra analyzer (Roche Diagnostics, Basel, Switzerland). Circulating levels of insulin, 25OHD, TSH, LH, FSH, prolactin, free thyroxine, free triiodothyronine and estradiol were determined using the ADVIA Centaur XP Immunoassay System (Siemens Healthcare Diagnostics, Munich, Germany) with chemiluminescence testing methodology using advanced acridinium ester technology. Concentrations of adrenocorticotropic hormone (ACTH) and insulin-like growth factor-1 (IGF-1) were measured using solid-phase enzyme-labeled chemiluminescent immunometric assays (Immulite, Siemens, Munich, Germany). The homeostasis model assessment of insulin resistance (HOMA-IR) was calculated by dividing the product of fasting plasma glucose (mmol/L) and fasting plasma insulin (mU/L) by 22.5.

### 2.5. Statistical Analysis

All continuous outcome variables, presented as the mean ± standard deviation, were natural log transformed to approximate normality. Comparisons between both groups were performed using Student’s *t*-tests for independent samples. Student’s *t*-test for paired data was used to compare pre-treatment and post-treatment values of continuous variables in each of the treatment groups. Qualitative data were compared using the χ^2^ test. The strength and direction of relationships between the outcome variables were estimated using Pearson’s r-tests. A probability value (*p*) less than 0.05 was considered statistically significant.

## 3. Results

Before initiation of therapy, no significant differences were observed between the study groups with respect to age, smoking habits, the proportion of patients with type 2 diabetes and prediabetes, causes of subclinical hypothyroidism, body mass index, blood pressure, and daily dietary vitamin D intake (excluding vitamin D administered in capsule form) (Table 1).

Two patients in group 1 and one patient in group 2 were withdrawn from the study due to gastrointestinal adverse effects associated with metformin treatment, including abdominal pain, bloating, diarrhea, and a metallic taste in the mouth. In all cases, these adverse effects resolved following discontinuation of metformin. One additional patient from group 1 was withdrawn because of relocation abroad. Statistical analyses were performed on data from 55 patients (93%) who completed the study and met the criteria for adherence. According to the power analysis, the study had 86% power to detect the anticipated difference in the primary outcome.

At baseline, the groups were comparable with respect to all the measured biomarkers (Table 2). The metformin treatment reduced glucose, HOMA-IR, HbA_1c_, TSH, and FSH in both study groups. Only metformin administered in combination with vitamin D, but not metformin monotherapy, increased circulating 25OHD levels and resulted in reductions in LH and prolactin levels. Concentrations of ACTH, free thyroid hormones, estradiol, and IGF-1 remained unchanged throughout the study. Post-treatment values of 25OHD, glucose homeostasis markers, TSH, and gonadotropins were higher in group 1 than in group 2 (Table 2). The treatment had no effect on body mass index or blood pressure in either group.

The effect size in 25OHD, fasting glucose, HOMA-IR, HbA_1c_, TSH, gonadotropins and prolactin differed between the two groups, whereas the magnitudes of change in the remaining outcome measures were comparable (Table 3).

In metformin-naïve postmenopausal women with insulin resistance and non-autoimmune subclinical hypothyroidism participating in the parallel study, vitamin D increased plasma 25OHD levels and reduced HOMA-IR, while having no significant effect on the remaining parameters assessed (Table 4).

In both study groups, the TSH- and gonadotropin-lowering effects of metformin correlated with baseline concentrations of these pituitary hormones (TSH: group 1: r = 0.524 [*p* < 0.0001] group 2: r = 0.547 [*p* < 0.0001]; FSH: group 1: r = 0.492 [*p* < 0.0001] group 2: r = 0.522 [*p* < 0.0001]; LH: group 1: r = 0.501 [*p* < 0.0001] group 2: r = 0.471 [*p* < 0.0001]). The reduction in HOMA-IR was positively correlated with baseline 25OHD levels (group 1: r = 0.356, *p* = 0.0195; group 2: r = 0.347, *p* = 0.0204).

## 4. Discussion

The metformin monotherapy significantly reduced TSH and FSH levels. Although a reduction in LH concentrations was observed, it did not reach statistical significance, possibly due to less elevated baseline LH levels compared with FSH. In contrast, metformin alone had no effect on circulating prolactin or ACTH concentrations, which were within the high-normal and normal ranges, respectively. These findings support the concept that the drug lowers anterior pituitary hormone levels only when they are increased [4,5,6]. Positive correlations between baseline hormone levels and reductions in TSH and gonadotropins indicate that metformin’s effect is proportional to pituitary secretory overactivity, whereas the absence of correlations between hormone changes suggests independent modulation of anterior pituitary cell populations.

A key finding of the present study is that combination therapy with metformin and vitamin D demonstrated superior efficacy over the metformin monotherapy in modulating anterior pituitary hormone secretion. The combined regimen was associated with a greater reduction in TSH and FSH concentrations, and decreases in LH and prolactin were observed exclusively in the combination group. As baseline 25OHD levels were within the reference range, these effects are unlikely to result from correction of vitamin D deficiency, as previously reported in individuals with low vitamin D status [12]. Although treatment allocation was based on patient preference, the selection procedure minimized between-group differences in age, glycemic control, baseline 25OHD, and TSH concentrations. Furthermore, the observed differences between the combination therapy and metformin alone cannot be attributed to baseline disparities in other hormonal parameters, as initial values of all the outcome measures were comparable between the groups.

Importantly, the observed effects cannot be accounted for by a simple summation of the actions of metformin and vitamin D. Although exogenous vitamin D has been shown in some—but not all—previous studies to reduce TSH concentrations in patients with autoimmune thyroiditis, this effect has been attributed to improved thyroid function resulting from decreased inflammatory cell infiltration [26,27,28]. To preclude any confounding influence of thyroid autoimmunity, the present study was restricted to women with non-autoimmune thyroid hypofunction. Furthermore, no effects of vitamin D monotherapy were observed in a separate cohort of insulin-resistant women with subclinical hypothyroidism who were not treated with metformin or other glucose-lowering agents. Collectively, these observations support the presence of a pharmacodynamic interaction between the two agents.

The clinical implications of the present findings remain to be fully elucidated. Metformin combined with vitamin D appears to be well tolerated in individuals with adequate baseline vitamin D status, and routine pre-treatment assessment of circulating 25OHD concentrations may not be required. Preclinical data demonstrate that selective blockade of FSH using highly specific antibodies directly suppresses osteoclast activity, resulting in increased bone mass, and is additionally associated with improved cognitive performance [29]. In turn, elevated postmenopausal LH levels have been shown to enhance β-amyloid production in neuronal cells and to promote inflammatory and oxidative stress responses in glial cells, mechanisms implicated in neurodegenerative pathology [30]. Collectively, these observations suggest that metformin/vitamin D combination therapy may represent a rational therapeutic strategy in postmenopausal women with type 2 diabetes or prediabetes and markedly increased risk of osteoporosis or Alzheimer’s disease. The absence of between-group differences in free thyroid hormone concentrations, along with the neutral effect of treatment on free thyroxine and free triiodothyronine levels, indicates that metformin/vitamin D combination therapy does not adversely affect thyroid function. Accordingly, despite modulating thyrotroph secretory activity, this intervention does not appear to increase the need for levothyroxine therapy, which is not routinely indicated in asymptomatic, non-autoimmune subclinical hypothyroidism. Given that persistently elevated TSH concentrations predispose to thyroid enlargement and the development of non-toxic nodular goiter [31], our findings suggest that the protective effect against thyroid growth and nodular disease is more pronounced with combined metformin and vitamin D therapy than with metformin alone. Finally, metformin/vitamin D combination therapy may confer potential benefits in patients with pituitary adenomas secreting thyrotropin or gonadotropins.

Additional findings also warrant discussion. First, vitamin D administered as monotherapy improved insulin sensitivity. The relatively modest effect observed, compared with previous studies [32,33], may be attributed to normal baseline 25OHD concentrations in all the participants. Consistent with this explanation, the reduction in HOMA-IR showed a positive correlation with baseline 25OHD levels. Notably, vitamin D potentiated the effects of metformin on all the assessed markers of glucose homeostasis, despite metformin being chronically administered at a high daily dose. These findings suggest that vitamin D add-on therapy should be considered in metformin-treated postmenopausal women with diabetes or prediabetes who fail to achieve adequate metabolic control. Second, combination therapy with metformin and vitamin D may obscure or delay the diagnosis of thyroid hypofunction. Therefore, assessment of TSH levels prior to treatment should be considered, at least in patients presenting with symptoms suggestive of hypothyroidism. Third, the absence of changes in free thyroid hormone levels argues against either beneficial or detrimental direct effects of metformin, administered alone or in combination with vitamin D, on thyroid function in individuals with hypothyroidism of non-autoimmune origin. Fourth, the combination therapy does not improve ovarian function after menopause. Finally, high-normal mean prolactin concentrations are likely associated with mild thyroid hypofunction (up to 22% of patients with subclinical hypothyroidism develop hyperprolactinemia [34]). The modest reduction in prolactin levels observed in individuals receiving combination therapy, despite unchanged free thyroid hormone levels, provides additional evidence supporting independent effects of metformin on distinct pituitary cell populations.

The biological basis for the greater effectiveness of metformin combined with exogenous vitamin D, compared with metformin alone, remains uncertain. Our findings demonstrate that metformin does not affect circulating 25OHD concentrations, which is consistent with the results of a previous randomized, placebo-controlled trial [35]. This indicates that metformin neither interferes with the intestinal absorption of vitamin D nor affects its metabolism. Metformin is eliminated unchanged via the kidneys and has a low propensity for clinically relevant drug interactions, largely restricted to compounds sharing cation transport pathways [36]. To date, no pharmacokinetic interactions between metformin and vitamin D or its metabolites have been reported. Although metformin plasma concentrations were not assessed in the present study, alterations in metformin absorption, metabolism, or excretion do not provide a convincing explanation for the observed effects. A more credible hypothesis involves convergence of metformin and vitamin D signaling at the level of pituitary adenosine monophosphate–activated protein kinase (AMPK). Due to the absence of a blood–brain barrier, metformin tends to accumulate in the pituitary gland [37,38]. Within the pituitary, thyrotrophs and gonadotrophs exhibit particularly high AMPK expression, and AMPK mediates metformin-induced inhibition of gonadotropin secretion in rats [9]. Vitamin D and its active metabolite have been shown to increase AMPK expression in the brain, skeletal muscle, and adipose tissue [39,40,41]. Finally, AMPK has been implicated in mediating interactions between metformin and vitamin D in extrapituitary tissues [42,43]. Alternative explanations may include synergistic effects of metformin and vitamin D on tuberoinfundibular dopaminergic neurons or potentiation of the pleiotropic effects of metformin by exogenous vitamin D. However, there is currently no experimental or clinical evidence supporting these mechanisms.

Several limitations of this study should be acknowledged. Although the sample size exceeded the minimum required, the relatively small cohort and the nonrandomized design substantially limit the generalizability and clinical relevance of the results. The validity of the findings may be compromised by potential sources of bias, including participant selection, assignment of patients to groups based on their choice, data ascertainment, and uncontrolled confounding variables. Because the study population was characterized by low selenium status and sufficient iodine intake [44,45], it remains unclear whether similar outcomes would be observed in individuals residing in selenium-replete and/or iodine-deficient regions. While methodological measures were implemented to reduce the influence of diurnal, seasonal, and analytical variability, the potential effect of regression toward the mean cannot be entirely ruled out. Finally, the study design does not allow elucidation of the underlying biological mechanisms responsible for the observed associations.

## 5. Conclusions

Although the study groups were matched for age, glucose concentration, plasma 25OHD, and TSH levels, the effects of metformin on indices of glucose homeostasis and circulating concentrations of TSH, gonadotropins, and prolactin in postmenopausal women with non-autoimmune subclinical hypothyroidism and adequate vitamin D status were more pronounced among those receiving concomitant exogenous vitamin D. These pituitary effects, whether observed with combination therapy or metformin monotherapy, were not accompanied by measurable changes in the function of peripheral endocrine organs (the thyroid gland and ovaries). Collectively, these observations indicate that patients presenting with markedly elevated anterior pituitary hormone concentrations may derive benefits from the combined administration of metformin and vitamin D, irrespective of baseline vitamin D status; however, this therapeutic approach does not appear to constitute an alternative to hormone replacement therapy in symptomatic individuals. Larger, well-designed randomized controlled trials are warranted to substantiate our findings, and further studies are needed to elucidate the molecular basis of metformin-vitamin D interactions.

## Figures and Tables

**Table 1:** Baseline characteristics of postmenopausal women with subclinical hypothyroidism and normal vitamin D status enrolled in the study.

Variable	Group 1 *	Group 2 **	*p*-Value
Number (*n*)	25	30	-
Age (years)	64 ± 8	65 ± 9	0.6680
Smokers (%)/number of cigarettes a day (*n*)/duration of smoking (years)	44/11 ± 6/30 ± 18	46/9 ± 7/34 ± 19	0.5915
Type 2 diabetes/prediabetes (%)	56/44	60/40	0.8382
Thyroid surgery/previous radioiodine therapy/thyroid agenesis or dysgenesis/genetic defects of thyroid hormone synthesis (%)	56/12/16/16	60/13/20/7	0.2462
Body mass index (kg/m^2^)	26.0 ± 4.9	25.8 ± 5.5	0.8884
Systolic blood pressure (mmHg)	131 ± 16	132 ± 15	0.8121
Diastolic blood pressure (mmHg)	85 ± 8	85 ± 6	1.0000
Vitamin D intake with food [µg/day]	16.0 ± 6.2	16.4 ± 7.8	0.8365

Unless otherwise indicated, values are reported as mean ± standard deviation. * metformin/vitamin D combination therapy; ** metformin monotherapy.

**Table 2:** The impact of treatment on the assessed outcome variables in the two patient cohorts.

Variable	Group 1 *	Group 2 **	*p*-Value
25OHD (nmol/L) [75–150]			
Prior to treatment	110 ± 21	114 ± 24	0.5179
Following treatment	140 ± 20	112 ± 19	**<0.0001**
*p*-value (before vs. following)	**<0.0001**	0.7217	-
Fasting glucose (mmol/L) [3.9–5.5]			
Prior to treatment	6.67 ± 0.61	6.56 ± 0.83	0.5846
Following treatment	5.22 ± 0.56	5.67 ± 0.78	**0.0194**
*p*-value (before vs. following)	**<0.0001**	**0.0001**	-
HOMA-IR [<2.0]			
Prior to treatment	4.6 ± 1.8	4.8 ± 2.0	0.7008
Following treatment	2.3 ± 1.4	3.1 ± 1.4	**0.0396**
*p*-value (before vs. following)	**<0.0001**	**0.0003**	-
HbA_1c_ (%) [<5.7]			
Prior to treatment	6.8 ± 0.6	6.7 ± 0.6	0.5409
Following treatment	5.3 ± 0.7	5.8 ± 0.7	**0.0109**
*p*-value (before vs. following)	**<0.0001**	**<0.0001**	-
TSH (mU/L) [0.4–4.5]			
Prior to treatment	7.5 ± 1.5	7.4 ± 1.3	0.7921
Following treatment	4.9 ± 1.9	6.2 ± 1.6	**0.0080**
*p*-value (before vs. following)	**<0.0001**	**0.0023**	-
FSH (U/L) [>30]			
Prior to treatment	81 ± 30	78 ± 28	0.7032
Following treatment	49 ± 18	61 ± 20	**0.0244**
*p*-value (before vs. following)	**<0.0001**	**0.0089**	-
LH (U/L) [>15]			
Prior to treatment	51 ± 25	53 ± 20	0.7430
Following treatment	35 ± 16	47 ± 24	**0.0375**
*p*-value (before vs. following)	**0.0097**	0.2972	
Prolactin (ng/mL) [2–20]			
Prior to treatment	20.3 ± 7.0	19.7 ± 6.8	0.7429
Following treatment	15.4 ± 5.8	17.5 ± 7.1	0.2413
*p*-value (before vs. following)	**0.0098**	0.2253	**-**
ACTH (pg/mL) [15–75]			
Prior to treatment	36 ± 18	40 ± 20	0.4432
Following treatment	41 ± 22	43 ± 20	0.7256
*p*-value (before vs. following)	0.3835	0.5635	-
Free thyroxine (pmol/L) [10.0–21.3]			
Prior to treatment	14.2 ± 3.2	14.0 ± 2.8	0.8057
Following treatment	15.4 ± 2.9	15.1 ± 3.4	0.7292
*p*-value (before vs. following)	0.1711	0.1765	-
Free triiodothyronine (pmol/L) [2.3–6.5]			
Prior to treatment	3.5 ± 0.7	3.4 ± 0.7	0.6000
Following treatment	3.8 ± 0.9	3.6 ± 0.8	0.3870
*p*-value (before vs. following)	0.1946	0.3070	-
Estradiol (pg/mL) [<30]			
Prior to treatment	15 ± 9	16 ± 8	0.6645
Following treatment	18 ± 8	17 ± 8	0.6463
*p*-value (before vs. following)	0.2189	0.6301	-
IGF-1 (ng/mL) [70–190]			
Prior to treatment	116 ± 35	120 ± 41	0.7123
Following treatment	123 ± 42	130 ± 46	0.5614
*p*-value (before vs. following)	0.5251	0.3778	-

Values are reported as mean ± standard deviation, with statistically significant changes denoted in bold. Reference values for postmenopausal women are indicated in square brackets. * metformin/vitamin D combination therapy; ** metformin monotherapy.

**Table 3:** Percentage changes from baseline in the outcome variables in both study groups.

Variable	Group 1 *	Group 2 **	*p*-Value
Δ 25OHD	27 ± 15	−2 ± 8	**<0.0001**
Δ Fasting glucose	−22 ± 8	−14 ± 6	**0.0001**
Δ HOMA-IR	−50 ± 23	−35 ± 19	**0.0106**
Δ HbA_1c_	−22 ± 8	−13 ± 7	**<0.0001**
Δ TSH	−35 ± 14	−16 ± 11	**<0.0001**
Δ FSH	−40 ± 18	−22 ± 12	**<0.0001**
Δ LH	−31 ± 16	−11 ± 10	**<0.0001**
Δ Prolactin	−24 ± 20	11 ± 15	**0.0081**
Δ ACTH	14 ± 34	8 ± 40	0.5561
Δ Free thyroxine	8 ± 11	8 ± 8	1.0000
Δ Free triiodothyronine	9 ± 12	6 ± 10	0.3164
Δ Estradiol	20 ± 48	6 ± 47	0.2379
Δ IGF-1	6 ± 20	8 ± 28	0.7661

Values are reported as mean ± standard deviation, with statistically significant changes denoted in bold. * metformin/vitamin D combination therapy; ** metformin monotherapy.

**Table 4:** The impact of vitamin D treatment on the assessed outcome variables in metformin-naïve postmenopausal women participating in the parallel study.

Variable	Prior to Treatment	Following Treatment	*p*-Value
25OHD (nmol/L)	108 ± 18	136 ± 22	**<0.0001**
Fasting glucose (mmol/L)	6.22 ± 0.50	6.00 ± 0.61	0.1695
HOMA-IR	4.3 ± 1.7	3.4 ± 1.0	**0.0210**
HbA_1c_ (%)	6.1 ± 0.5	6.0 ± 0.5	0.4829
TSH (mU/L)	7.3 ± 1.2	7.5 ± 1.6	0.6194
FSH (U/L)	84 ± 35	80 ± 31	0.6708
LH (U/L)	47 ± 18	52 ± 24	0.4563
Prolactin (ng/mL)	18.3 ± 7.5	18.9 ± 7.8	0.7828
ACTH (pg/mL)	42 ± 19	40 ± 17	0.6966
Free thyroxine (pmol/L)	14.4 ± 2.6	14.1 ± 2.9	0.7018
Free triiodothyronine (pmol/L)	3.4 ± 0.7	3.4 ± 0.8	1.0000
Estradiol (pg/mL)	19 ± 7	18 ± 8	0.6402
IGF-1 (ng/mL)	118 ± 35	124 ± 40	0.5751

Values are reported as mean ± standard deviation, with statistically significant changes denoted in bold.

## Data Availability

The data presented in this study are available on request from the corresponding author due to privacy.
